# Turbulence-induced droplet grouping and augmented rain formation in cumulus clouds

**DOI:** 10.1038/s41598-024-61036-z

**Published:** 2024-05-04

**Authors:** Siddharth Gumber, Sudarsan Bera, Satyajit Ghosh, Thara V. Prabhakaran

**Affiliations:** 1 Atmospheric, Ice and Climate Team, British Antarctic Survey, Cambridge, CB3 0ET UK; 2 St. Edmunds College, University of Cambridge, Cambridge, CB3 0BN UK; 3 Indian Institute of Tropical Meteorology, Ministry of Earth Sciences, Government of India, Pune, India; 4 School of Mechanical Engineering, Vellore Institute of Technology, Vellore, 632014 India; 5 School of Earth and Environment, University of Leeds, Leeds, UK

**Keywords:** Microscale vortices, Accretion, Auto-conversion, Droplet settling velocity, Large eddy simulation, Atmospheric science, Fluid dynamics

## Abstract

This paper provides the first observational analysis of how droplet separation is impacted by the flinging action of microscale vortices in turbulent clouds over a select radii range and how they vary over cloud cores and along the peripheral edges. It is premised that this mechanism initiates droplet separation within a cloud volume soon after condensational growth, largely in the cloud core, and operates until the cloud droplet radii exceed 20–30 µm when this effect fades rapidly. New observations are presented showing how microscale vortices also impact the settling rates of droplets over a critical size range (6–18 µm) causing them to sediment faster than in still air affecting swept volumes and thereby impacting the rain initiation and formation. Large-scale atmospheric models ignore these microscale effects linked to rapid droplet growth during the early stages of cloud conversion. Previous studies on droplet spatial organization along the cloud edges and inside the deep core have shown that homogeneous Poisson statistics, indicative of the presence of a vigorous in-cloud mixing process at small scales obtained, in contrast to an inhomogeneous distribution along the edges. In this paper, it is established that this marked core region, homogeneity can be linked to microscale vortical activity which flings cloud droplets in the range of 6–18 µm outward. The typical radius of the droplet trajectories or the droplet flung radii around the vortices correlates with the interparticle distance strongly. The correlation starts to diminish as one proceeds from the central core to the cloud fringes because of the added entrainment of cloud-free air. These first results imply that droplet growth in the core is first augmented with this small-scale interaction prior to other more large-scale processes involving entrainment mixing. This first study, combining these amplified velocities are included in a Weather Research and Forecasting- LES case study. Not only are significant differences observed in the cloud morphology when compared to a baseline case, but the ‘enhanced’ case also shows early commencement of rainfall along with intense precipitation activity compared to the ‘standard’ baseline case. It is also shown that the modelled equilibrium raindrop spectrum agrees better with observations when the enhanced droplet sedimentation rates mediated by microscale vortices are included in the calculations compared to the case where only still-air terminal velocities are used.

## Introduction

Particle-laden flows in turbulent fluids have diverse applications in several disciplines—aerospace and chemical engineering, manufacturing engineering and almost every stream of fluid dynamics. In this era of the Anthropocene, it is imperative to link fundamental fluid dynamical studies to engineering and applied sciences to explore causes and seek solutions for outstanding meteorological problems including the scourge of global warming. This paper explores microscale (~ ${10}^{-3} {\text{m}}$) vortical interactions within viscous fluid flows and shows through modelling and observational analyses how this may yield a better understanding of some complex and partially understood cloud processes. Under-represented cloud processes pose the biggest challenge in weather predictions and thus are tenuously linked to all attempts to forecast weather and climate over a warming planet so that attempts to conduct large-scale technological interventions to counter the impact of global warming can be supported by newer modelling insights^1–3^.

Several efforts have been made all over the world to understand how rain formation is triggered in deep convective clouds^4–6^ [references therein]. Past research focussed on several mechanisms including turbulence-induced mechanisms and a definitive summary was given by Jonas^7^. Research within the Indian sub-continent has an added relevance—India is a monsoon-dominated country and millions of vulnerable citizens depend on monsoonal rains for their livelihoods. The Government-led Indian Institute of Tropical Meteorology (IITM) in Pune pioneered cloud microphysical research and strengthened modelling as well as observation-based research and an overview can be found in Kulkarni et al.^8^. The IITM also pioneered engineered interventions through cloud seeding operations in rain-starved districts that involved international collaborations with leading cloud microphysicists worldwide^9,10^. Several categories of research avenues were classified to explain how from the initial condensation-driven cloud droplet growth, large raindrops form, and a good summary is presented in Grabowski and Wang^11^. Theme-wise examples include the intermittent supersaturation fluctuation by turbulent updraughts^12–15^, the processes associated with droplet inhomogeneities along with preferential concentration distributions, entrainment mediated mixing of environmental air with cloud air^16–18^, and even how giant cloud condensation nuclei quickly yield drizzle drops^19,20^, and lastly the various mixing processes that result in spectral broadening of DSDs^17,21–23^.

**Figure 1:**
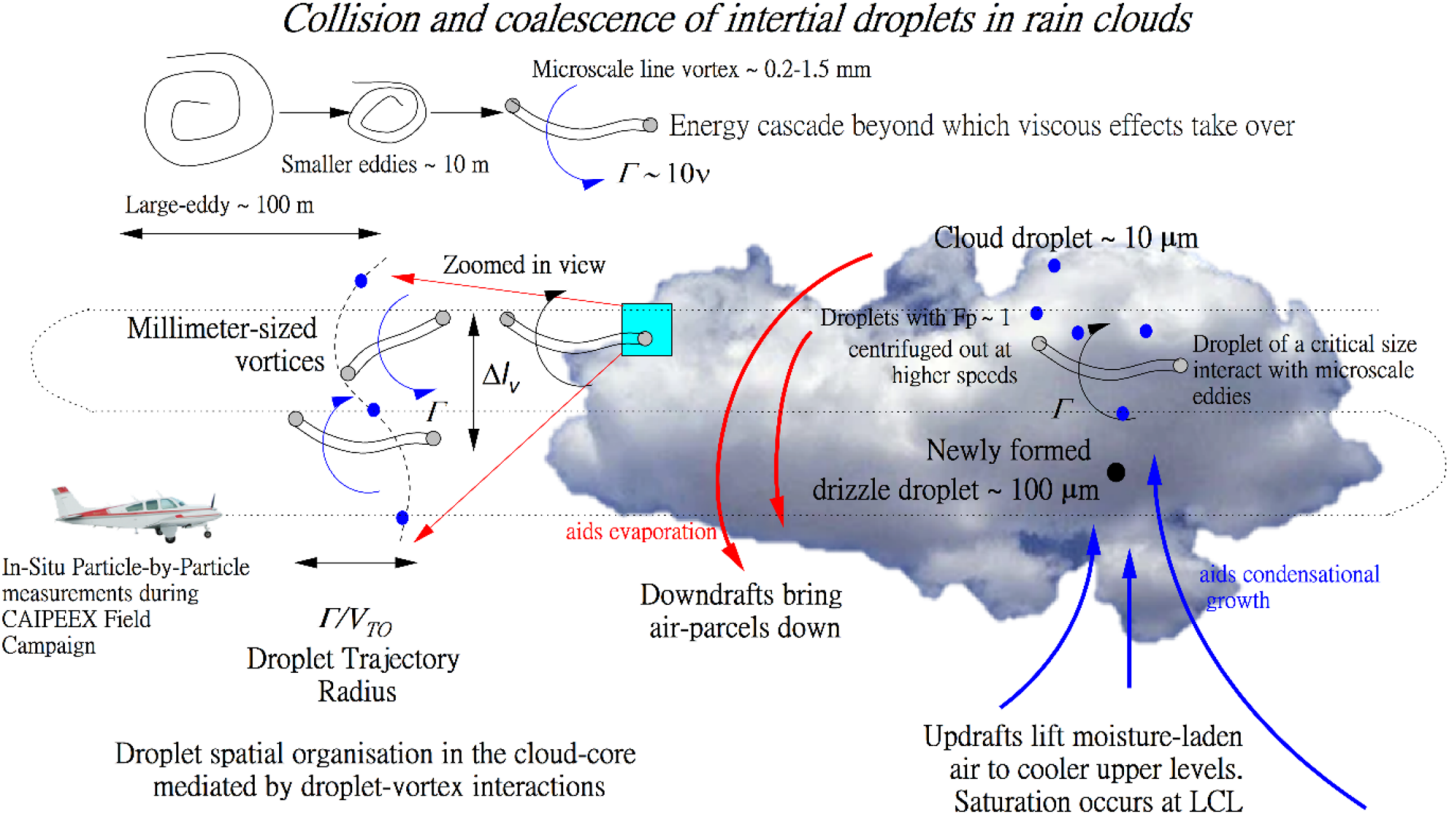
Schematic diagram illustrating various microscale processes in a developing cumulus cloud. The zoomed-in window (cyan color) shows how droplets of all sizes interact with rotating line vortices within the cloud. Smaller droplets are flung outwards so that their settling rates are enhanced. The Cloud Droplet Probe (CDP) onboard the IITM’s research aircraft captures information on inter-droplet spacings as well as on the spatial organisation of microscale vortices within the cloud.

Microscale eddies are ubiquitously present in all types of fluid flows and their interactions pose novel challenges in quantifying the effect of suspended particles and their settling rates. Dávila & Hunt (2001) and Ghosh et al. describe the quantification of drift integrals (essentially the added length scale a falling particle must negotiate to settle past a microscale eddy)^24,25^. Contemporary research continues to use these calculations applying them to diverse fluid mechanical applications^26^ and a review can be found from Shaw^27^. However, this new mechanism required supporting experimental data in the form of fast response in situ data to study how interparticle distances are modulated by such droplet-vortical interactions. The separation and hence their starting co-ordinates determine how droplets with differing radii are deflected from their initial straight trajectories to curved lines bringing them closer for collision and coalescence processes (see Fig. 1). This first paper attempts to do this.

We first proceed with a brief description of the physics of such interactions. As early as 1963, Csanady reported the ‘Crossing trajectory effect’ wherein sedimenting droplets encounter a bias in their trajectories moving them towards regions of downward fluid motion around vortices resulting in increased settling over a range of particle relaxation times ${\tau }_{p}$^24,28^. Subsequently, even DNS simulations by Ayala^29^ confirmed that the turbulent settling velocities of droplets with radii less than 40 µm are greater than their settling velocities in still air, and when $r$ $\cong$ 6–10 µm, one encounters the largest increase in settling velocity—this was explained fully by Dávila & Hunt (2001) for inertial particles settling near a Rankine vortex^24,30^. They derived a particle Froude number independent of ε, the TKE dissipation rate. For typical cloud conditions, ${F}_{p}$ ~ 1 for droplets with $r$ ~ 10–20 µm. The enhancement of the settling velocity reaches a maximum for $r$ ~ 6–10 µm regardless of the value *ε*. This impacts the preferential sweeping of droplets so that cloud droplets can grow rapidly from the critical radius of 20 µm to well over 50–80 µm^25^. The effect is most pronounced when cloud droplet radii are in the range of (6–18 µm) and rapidly fade with increasing radii. Convective clouds indeed have a large number concentration of droplets with this size range^31^.

In this paper, this microscale effect is applied to a cloud simulation to explore cloud auto-conversion rates induced by these effects. We illustrate that microscale vortices (MSVs) not only have a large spatial extent in monsoonal convective clouds, but they also impact conversion rates. Cloud conversion typically proceeds when some droplets exceed the critical radius threshold of 20 µm^25,32,33^. For droplets smaller than this critical size necessary for auto-conversion, the settling rates are impacted by the ubiquitous presence of microscale vortices. We show that the modulating effects of such vortices are always present in varying degrees- in clouds with low as well as high levels of turbulence, in both upward and downward moving parts with toroidal effects^34–38^ [references therein] and when the cloud boundaries are entraining air and cover the entire fabric of the fluid flow. The droplets revert to their original fall velocities when their radii exceed 20 µm. In essence, the amplification effect fades when the droplets are too large so that the vortical rotation does not impact the settling rates but continues to operate as long the embedded droplets have radii within this critical size range. Large eddy simulations and even large-scale global climate models (GCMs) use power law relations to quantify the fall velocity of cloud droplets which are size-dependent but do not include this amplification effect^39,40^.

From the preceding paragraph, it is seen that most of the suggested mechanisms largely dealt with entrainment and mixing processes and preferential concentration mechanisms to explain the rapid growth of cloud droplets. There are also several papers suggesting the role of large-scale turbulence in enhancing rapid collision growth (turbulence-induced collision enhancement)^4,29,41–43^. The remit of this paper however concerns the application of in situ droplet scale measurements and their interactions with micro-scale processes and not large-scale processes which have been published as indicated. In a convective cloud, and most monsoon clouds, it is expected that the cloud droplet terminal velocity (in still air) is less than the root-mean-square fluid velocity^44^. In such a situation, as described earlier, the actual fall speed of particles with finite inertia will be amplified from interactions with the fluid turbulence mediated by MSVs^24,25,27^. Ghosh & Jonas provided an analytical formulation to capture this fall velocity enhancement and applied these results for the growth of a large drop falling through a population of smaller cloud droplets and showed that the Baker calculation can be retrieved as a limiting case when the MSV amplification is set to zero^45,46^. The role of microscale vortices impacting settling rates of small droplets has been an active domain of investigation in many fluid mechanical applications. Since the publication of Davila and Hunt^24^, who provided a sound theoretical basis for such enhancements to occur over a critical size range, earlier models used the Davila and Hunt theory in formulating analytical codes and in presenting new droplet growth rates^24,25^. Subsequently, Shaw in his definitive review paper have discussed this mechanism with several other large-scale processes that operate within clouds^27^. There are existing theories as propounded by these earlier studies; however, for the lack of in-situ fine-scale observational datasets in the developmental stages of clouds, these theories could not be used to study droplet groupings causing small cloud droplets to settle faster than their Stokesian velocities. The success of the CAIPEEX Mission urged us to revisit these earlier findings. The most rigorous observational evidence using this very dataset showed a great deal of variability of droplet IPDs within different parts of the cloud, i.e. core and edges. It has been shown how this signature is clearer in the core where droplets are well-shielded from other extraneous mechanisms such as entrainment. Importantly, even the most widely used WRF model uses still-air settling rates. This is possibly the first study where these new results are used in WRF Microphysics. This paper for the first time has used a very extensive aircraft-based in-situ data to first show how the droplet inter-particle distances (IPD) are modulated and thereafter how this reorganised grouping alters cloud conversion processes. It is emphasized that while large scale processes (up and downdraughts) move air-parcels around, microscale processes are expected to operate within parcels independently even as they are translated spatially.

Over the years instrumented aircraft-based observations at very high resolution and frequency developed substantially. The IITM in Pune pioneered such observations and for the first time, observational datasets (described in “Methods” section) were used to correlate droplet interparticle distances with estimated droplet trajectory radii (we call this the flung radius) as they approached line vortices. This is important because droplet groupings in the cloud core determine subsequent processes related to self-collection and auto-conversion of cloud water to rainwater. To garner statistically significant inferences to relate microphysical attributes of vortical distributions vis-à-vis droplet grouping, it is essential to have (i) multiple cloud passes at high frequencies so that droplet arrival times are accurately obtained (ii) to repeat observations at varying spatial scales (iii) to record droplet sizes and numbers over both upstreaming and down streaming parts of a towering cloud.

In what follows, we first describe briefly how steps (i)–(iii) were completed by Bera et al.^21^. They describe the statistical and microphysical properties of liquid cloud droplets (with diameter 2–50 µm) in the growing stage of cumulus clouds illustrated by in situ Particle-by-Particle (PbP) measurements. This is described in “Methods” section. Then we describe the generic conditions for the synoptic development of a monsoon cloud event (our Case Study). This cloud had developed drizzle rain drops in the upper reaches and in-cloud observations were chosen at these levels where the hydrometeors fell through varying levels of turbulence, spatially as well as temporally. Rain amounts are then processed from cloud conversion. The latter is largely sourced from cloud droplets spread over small to large radii (5–20 µm). This is followed by the section MSV Mediated velocity amplification of cloud droplets (“MSV mediated velocity amplification of cloud droplets” section). In “Results from an LES case study” section deals with the incorporation of amplified settling velocities in an LES cloud model and singling out the induced effect with a comparison where only still air fall velocities are used. In “Conclusions” section describes Wider Implications.

## Methods

### Data and instruments details

The in-situ flight observations used in this study are ideally suited for this analysis and are sourced from the extensive field experiment known as “Cloud Aerosol Interaction and Precipitation Enhancement Experiment (CAIPEEX)”^8,21^ during phase IV (2018–2019) over peninsular India. CAIPEEX-IV was customised to cover the rain-shadow region of the Western Ghats, over Solapur city (17.68 $^\circ {\text{N}}$, 75.92 $^\circ {\text{E}}$) considered a lean region. Instruments on board a Beechcraft-200 aircraft were used for the microphysical characterisation of convective monsoonal clouds. CDP-2 (developed by Droplet Measurement Technologies LLC, USA) is used for cloud droplet measurement which has K-tips to minimize the droplet shattering effect. This instrument recorded Particle-by-Particle data of each cloud droplet. Due to its data storage capacity, it undertakes PbP measurements for the first 256 droplets. The sophisticated instrumentation on board recorded cloud attributes of each cloud droplet (spanning a diameter range of 2–50 µm) and microphysical data was available from the dataset PbP (Particle-by-Particle) encompassing cloud penetrations over horizontal legs at varying altitudes with a detailing also of particle arrival times. This observational dataset is unique as it can reveal cloud microphysical attributes at much smaller scales (< 1 ${\text{mm}}$) allowing us to explore droplet features and their interactions with small-scale turbulence (or MSV’s) at very-high spatial resolutions. Particle-by-Particle measurements collocated each second constitute less than 6% of the total droplets probed by the instrument (due to its storage capacity) but have been benchmarked with 1 ${\text{Hz}}$ observations presented by Bera et al.^21^. This comparison of droplet size spectra (DSDs) from the PbP as well as from the 1 ${\text{Hz}}$ datasets lent an added level of reliability assuring us that the PbP gave statistically representative measurements^21^. CDP-2 measures all particles coming in the depth of the field (DOF) of the laser beam which has a small cross-section of about 0.267 ${{\text{mm}}}^{2}$. CDP-PbP has a very high spatial resolution (≅ 0.0023 ${\text{mm}}$ at aircraft speed of 90 ${{\text{ms}}}^{-1}$) set by a 40 ${\text{MHz}}$ instrument clock. Size resolution ($\Delta D$) of CDP is 1 µm for droplet diameter ($D$) in the range 3–14 µm and 2 µm for diameters spanning 16–50 µm. Measurement uncertainties/biases in CDP-2 are further discussed in Bera et al.,^21^. There are several sources of uncertainties/biases which may arise such as: heterogeneous sizing response due to laser misalignment; coincidence effect; uncertainty associated with the calibration data that is used for droplet sizing. In PbP dataset, the spatial heterogeneity of droplets may lead to overestimation or underestimation in droplet concentration value. PbP data may get biases in droplet sizing if not properly calibrated. To control this, a regular calibration of the probe was undertaken during the CAIPEEX experiment to ensure the laser alignment was proper. Additionally, droplet coincidence effect may result in over-sizing and under-counting of droplets^47^. CDP-2 gives PbP data which has minimum coincidence artefacts (< 5%) as estimated by Lance et al.^48^.

For measuring larger drizzle-sized drops in the diameter range 100–6200 µm in the clouds, a Precipitation Imaging Probe (PIP-DMT) was operated at 1 ${\text{Hz}}$ sampling frequency during the experiment. The Aircraft’s Integrated Meteorological Measurement System (AIMMS-20) was additionally used to measure the air temperature, relative humidity, vertical velocity ($w$) and the aircraft’s position (latitude, longitude, and altitude). The AIMMS-20 system was used to conduct wind measurements ($u$, $v$, $w$) at a high resolution of 20 ${\text{Hz}}$ revealing a spatial scale of nearly 5 $m$ and the data was utilized for estimating cloud turbulent kinetic energy (TKE). The instrument accuracy and resolution for wind estimation were 0.50 ${\text{m}}/{\text{s}}$ (at a True Air Speed (TAS) of 75 ${\text{m}}/{\text{s}}$) and 0.01 ${\text{m}}/{\text{s}}$, respectively. All instruments used on-board the aircraft were factory calibrated before starting the experiment. Data quality control was achieved by periodic calibration of instruments, preventive maintenance, data screening, and data validation processes. Collected data were examined regularly after each flight and pre-processed by removing error data or artifacts and correcting data for known instrument biases. All data used in this study is pre-processed to reduce minimum bias/uncertainty. For the purposes of this analysis, several observational inferences very relatable to this study could be obtained.

**Figure 2:**
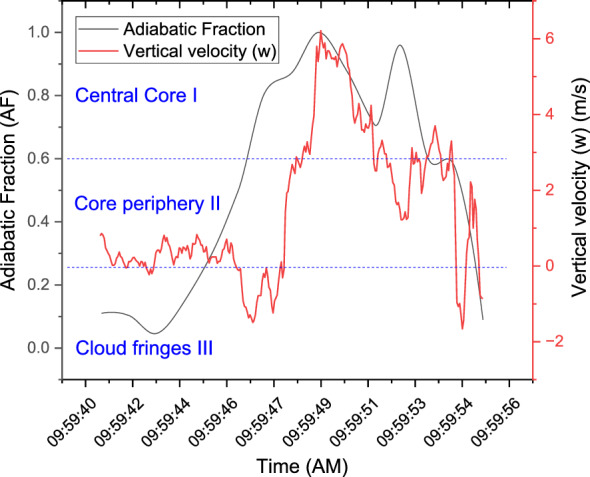
Temporal observations of vertical velocity and Adiabatic Fraction (AF) during a cloud penetration midway through the cloud at 3.2 ${\text{m}}$ . The droplet-rich regions (with high values of liquid water content) in the cloud core have been identified based on the adiabatic fraction (AF = $LWC/LW{C}_{ad}$) value of greater than 0.6 and vertical velocity ($w$) greater than 1 ${\text{m}}/{\text{s}}$.

Several cloud passes were made during the experiment, however, in this study, we have only considered cloud penetrations carried out at constant altitudes of 3.2, 4.3 ${\text{km}}$ in a towering cumulus cloud at its developing stage. It was noted that the 3.2 ${\text{km}}$ cloud pass was dominated by a preponderance of smaller droplets (no raindrops seen), however, larger drops (drizzle) were noted in the upper reaches at 4.3 ${\text{km}}$ as revealed by the PIP observations. Figure 2 shows the temporal observations of vertical velocity and Adiabatic Fraction (AF, the ratio of observed liquid water content to an adiabatic estimate of a cloud’s liquid water content) during a cloud penetration.

The droplet-rich regions (with high values of liquid water content) in the central cloud core have been identified based on the adiabatic fraction (AF = $LWC/LW{C}_{ad}$) value of greater than 0.6 and vertical velocity ($w$) greater than 1 $m/s$. The core-periphery Zone 2 has an AF value between 0.25 and 0.6 and also features positive vertical velocities (0 $<w<$ 1) and Zone 3, which is positioned along the cloud edges has been identified with AF less than 0.25 and negative updraught speeds ($w<0$). It is of much interest to explore the processes that govern droplet separation within these zones.

### Droplet separation and particle grouping statistics for a convective cloud

Processes that modulate the droplet’s size spectra and the associated rainfall mechanisms strongly depend on the mixing of differently saturated cloud regimes or the mixing of the environmental air with cloudy volumes^17,49^. However, mixing is scale dependent. Entrainment processes involve large length scales (hundreds of metres) whilst microscale mixing operates at the smallest millimetre scales of dissipating eddies. Due to the scarcity of finer-scale observations, mixing remains relatively less explored at scales down to a metre, and only some model-based understanding from direct numerical simulations illustrates these processes numerically^50–52^. The present observations (PbP) are therefore worthy and extremely useful for studying mixing-related processes vis-à-vis inter-droplet organisation at finer scales (∼${10}^{-3} {\text{m}}$).

**Figure 3:**
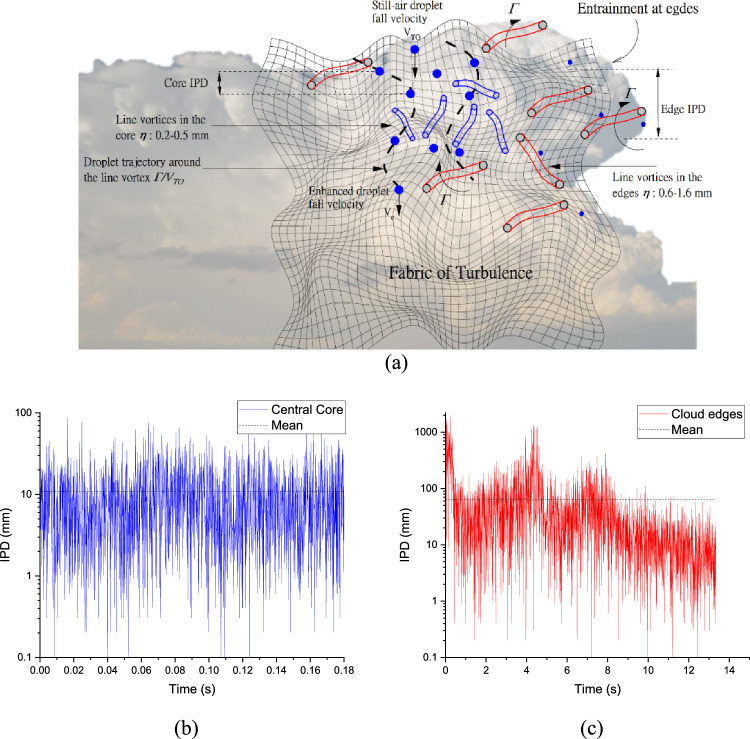
(**a**) Schematic showing how microscale line vortices are embedded within the fabric of turbulence. The central core subsumes larger droplets that interact with rotating line vortices. Note that ${V}_{TO}$ is the droplet’s still-air settling rate and ${V}_{e}$ is the droplet’s enhanced settling rate after it has apprehended a rotating vortex. Particle-by-particle measurements capture essential details on the droplet organisation within a cloud (**b**) Variation of the inter-particle distances of cloud droplets ($\lambda$*,* ${\text{mm}}$) in the central core and (**c**) Same as (**b**) but in the cloud-edge, during a time instance. Note that the Horizontal dashed lines indicate mean values of $\lambda$ which are 10.91 ${\text{mm}}$ and 63.13 ${\text{mm}}$ for the central core and edge, respectively.

The inter-arrival time ($\Delta t$) of individual droplets is used in the estimation of inter-particle distances (IPD) ($\lambda =\Delta t\times TAS$), where $TAS$ is the true airspeed. IPDs during a cloud pass at an altitude of 3.2 ${\text{km}}$ are presented in Fig. 3 and show a great deal of variability between what obtains in the shielded cloud core as opposed to the diluted edges.

Observations were collected over instances of 1 $s$ tracking the first 256 particles and marked along the *x*-axis. The inter-particle distance of two consecutive droplets varied from 0.1 to 100 ${\text{mm}}$ in the undiluted cloud core part and 0.1–1000 ${\text{mm}}$ in the diluted edges. The narrower inter-droplet separations in the core allow for the possibility of droplets coming closer more easily and we show in the next section how the presence of MSVs heightens this possibility. We show later that with a wide range of drop sizes greatly impacted by MSVs, the flinging action of vortices contributes largely to the observed homogeneous Poisson statistics in the Bera et al.^21^ study. It is hypothesized that the MSV effect promotes greater mixing so that even when an assortment of cloud droplets is homogeneously distributed, there can be rapid droplet growth causing spectral broadening. From the concept diagram shown in Figs. 1 and 3a, we show how the traced trajectories have different radii of curvatures as they descend past a vortex tube. The largest droplet is least affected by the presence of these stretched line vortices whilst the smallest droplet is most affected with a large flung radius. This vortex-mediated separation is a precursor to the interparticle distance as is evident from Fig. 12 where one observes a strong correlation between these two quantities in the central core. Further, Fig. 1 also pictorially depicts the concept of a drift integral—in essence, a summing up of all added curved lengths (as opposed to straight trajectories when they fall without the MSVs) that droplets negotiate because of the presence of vortices. The droplet size-dependent drift integrals can be both positive (when there is a lowering of velocities) and negative (when there is an amplification of velocities)^24,25^. Within the remit of this study, the drift integrals were negative because, in this convective cloud, droplet accumulation was high in downward fluid velocity regions^53^. The combined effect of viscosity and vortical circulation is expressed as a particle Froude number and it has been shown that large amplifications happen when ${F}_{p}$ ~ 1, as in this CAIPEEX study. In the following sections, we study the import of MSV effects across different cloud zones, i.e. over the less-diluted cloud core (almost entrainment-free), along the intermediately diluted zone as well as along the highly-diluted edges so that an unmistakeable MSV signature can be extracted. To understand the microphysical impact of MSV-mediated mixing across distinct spatial scales, mixing diagrams have been generated following Bera et al.,^21^. Lastly, amplified velocities based on droplet-eddy interactions have been hardwired in the Weather Research and Forecasting (WRF) model within the remit of a cloud auto-conversion scheme. We have further validated the modelled rain drop size distributions (accounting for these new effects) against direct measurements in the cloud at the initiation of raindrop formation and it is shown that the modelled rain-drop spectra agree better with observations compared to a baseline case where only still air values are used.

### Integrating MSV mediated velocity amplification in cloud processes

Mass transfer of cloud water into rainwater mediated by auto-conversion simply entails the processes of droplet amalgamation or droplet grouping. The cloud conversion rate is proportional to the cloud droplet fall-velocity times the droplet cross-sectional area and can be written as:

1 $$-\frac{dm}{dt}\propto V(r)\times \left(\pi {r}^{2}N\right)$$

Here, $m$ is the cloud mass $({\text{g}}{.{\text{m}}}^{-3})$, $N$ the cloud droplet number density (droplets ${{\text{m}}}^{-3})$, and $V(r)$ the velocity of the falling cloud droplets. It is therefore clear that the droplet fall velocity is a driving parameter for cloud and rain processes. Since the fall velocity is amplified over the critical size regime, this will directly impact cloud water conversion amounts. At this stage, it is important to discuss related research. Kessler^54^ initiated pioneering work to quantify rates of cloud water auto-conversion, and the versatility and robustness of his work is testified by the fact that his procedure is still incorporated within most microphysics schemes in Large Eddy and Cloud models including the WRF-ARW model. The process is described by the following simple formulation (Kessler)^54^:

2 $$-\frac{dm}{dt}={k}_{1}(m-a)$$

here, ${k}_{1}$ (${{\text{s}}}^{-1}$) is the auto-conversion rate, and $a$ (${{\text{gm}}}^{-3}$) the auto-conversion threshold. From Eqs. (1) and (2), one gathers that both equations attest to the same process of cloud water conversion to rainwater and an equivalence must be established between the operating parameters of both formulations. This is now described. Equation (2) can be written as:

3 $$- \frac{dm}{{dt}} = V\left( r \right)\left[ {N\pi \overline{{r^{2} }} } \right]E \times m = [k_{0} \overline{{r^{2} }} \left] {\overline{ E} } \right[N\pi \overline{{r^{2} }} ] m{\mathbf{\mathbb{Z}}}$$

here, $k_{0}$ is $1.2 \times 10^{8} m^{ - 1} s^{ - 1}$. A further simplification yields the following:

4 $$- \frac{dm}{{dt}} = \pi k_{0} 0.55N\overline{{r^{4} }} m \times H\left( {r - 10{\mu m}} \right)$$

Moreover, clouds are stable up to a certain threshold of the cloud mass $(a)$ or a certain droplet size after which a certain fraction of the cloud mass converts to rain mass $(M)$. A Heavyside function is introduced in Eq. (4) so that once the cloud droplet exceeds a critical size of 10 $\mu m$, cloud conversion into rain can take place. Equation (2) can be re-written to obtain Eq. (4), where ${k}_{1}=\pi {k}_{0}0.55N\overline{{r }^{4}}$ or ${k}_{1}=V(r)\left[\pi N\overline{{r }^{2}}\right]E.$ One notices that the Kessler formulation’s easy incorporation stems from the fact that there are only two adjustable parameters that modellers can use- the auto-conversion rate ${k}_{1}$, typically ~${10}^{-3}$ ${{\text{s}}}^{-1}$ and chosen auto-conversion thresholds (vary between 0.1 and 1 ${{\text{gm}}}^{-3}$). We now proceed to discuss how this process of cloud conversion might be impacted by in-cloud turbulence at micro-scales. From Eq. (4), it is amply clear that the fall velocity of the cloud droplets has a major impact on the auto-conversion process. If the assumed values are simply the still air fall velocities (as is assumed by all models including the WRF-ARW) errors shall creep in the quantification of the auto-conversion rates. Addressing this deficiency forms an important part of this paper. Before we set out to formulate the necessary changes to re-compute the corrected auto-conversion rates, it is worthwhile to briefly review some important papers that have the Kessler formalism as their basis.

Earlier studies have used Kessler-type auto-conversion formulations which assume a *Stokesian* velocity dependence for the falling cloud droplets^45,55–60^. However, more recent studies by Ghosh & Jonas, Dávila & Hunt (2001), and Ghosh et al. have shown that the ratio of the actual fall velocity to the *Stokesian* terminal velocity over a critical radius range ($r$ less than 20 $\mathrm{\mu m}$) is greater than unity, with a large effect for the smallest sizes and fading progressively for larger droplets with this ratio asymptotically reaching the value of unity^24,25,45^. In this paper, we have accounted for the size dependence of the amplification effect (the actual fall velocity in the turbulent flow field normalised over the still air fall velocity of the droplet). The auto-conversion term can thus be written as a function of the modified fall velocity as:

5 $$-\frac{dm}{dt}={V}_{enh}\left(r\right) \left[\pi N\overline{{r }^{2}}\right]E\times m H(r-10\mu m)$$

A further simplification yields the following:

6 $$-\frac{dm}{dt}=\pi {k}_{0}0.55N\overline{{r }^{4}} \left(1-\alpha D\right)\times m H\left(r-10\mu m\right)$$

The modified auto conversion rate ${k}_{1}{\prime}$ from Eq. (6) can be written as: ${k}_{1}{\prime}={V}_{enh}(r)$ $\left[\pi N\overline{{r }^{2}}\right]E$ or ${k}_{1}{\prime}={k}_{0}{r}^{2}(1-\alpha D)$ $\left[\pi N\overline{{r }^{2}}\right]E.$ For the baseline case, when the amplification effects are ignored i.e., $D\to 0$, the modified auto-conversion rate $({k}_{1}{\prime})$ approaches ${k}_{1}$ as shown earlier (standard Kessler auto-conversion rate)^45,54,61^. Thus, a simplified expression for the cloud conversion rate which accounts for enhanced droplet settling modulated by microscale vortices is given by Eq. (6).

### WRF modelling

The Weather Research and Forecasting (WRF)-LES model (v3.8.1) is a state-of-the-art atmospheric simulation suite designed for numerical weather prediction for both operational as well as research applications^62^. The suite contains fully compressible, non-hydrostatic, Navier–Stokes equations within an *Eulerian* framework. The model uses Arakawa C-grid spatial staggering in the horizontal direction and allows for a higher order *Runge–Kutta* integration temporally. Additionally, the suite provides the user with several choices of physics schemes to explicitly resolve cloud as well as precipitation processes based on the intended application. In order to explicitly resolve the energy-containing eddies over Solapur, it was deemed appropriate to configure the WRF in the large eddy mode in a manner outlined earlier by Seifert et al. & Hoffmann et al.^4,63^. The spatial resolution was fixed at 50 $\times$ 50 ${\text{m}}$ in the horizontal and 100 $m$ in the vertical covering a control volume with dimensions 10 $\times$ 10 $\times$ 5 ${\text{km}}$ This version of the WRF model was tested for grid independent sensitivity and convergence tests where it was shown that grid-independence was achieved^64^. This methodology enabled simulations free from numerical biases arising from the choice of grid size. Earlier LES studies have assumed various resolutions to ascertain the optimal element size range. Studies have shown that a horizontal resolution range of 100–200 ${\text{m}}$ is sufficient for simulating city-scale boundary layer attributes^65^ [references therein]. Thomas et al.^65^ highlighted that if the aim is simulating boundary layer attributes, one may select a slightly coarser resolution of 200 m, but if the objective is to simulate cloud-eddy structures, a grid size of ~ 100 m should be sufficient. Based on essential stipulations provided by these earlier studies, the horizontal and vertical resolutions in the present study were fixed at 100 m and 150 m (average between two consecutive vertical levels), respectively, to be within the grid-independent dynamics as desired. The same model was also validated for rain amounts in an earlier study by Gumber and Ghosh^66^. A smaller time-step of 0.3 $s$ was required to achieve numerical stability. Sub-grid scale turbulence characterisation was done through a 1.5-order TKE closure scheme which uses prognostic equations to characterise the eddy viscosity coefficients. The LES was configured with weather soundings (radiosonde observation) including vertical profiles of the potential temperature $(\theta )$, the relative humidity, $u$ and $v$ winds over Solapur for 20th July 2018 05:39:26 UTC.

## Results and discussions

### MSV mediated velocity amplification of cloud droplets

**Figure 4:**
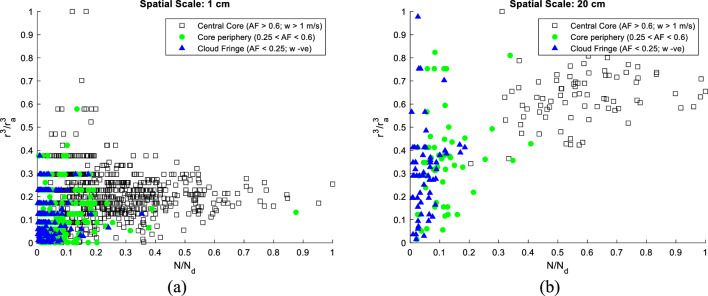
Microphysics diagram at two spatial scales (**a**) 1 ${\text{cm}}$ (**b**) 20 ${\text{cm}}$ using PbP data for a cloud penetration altitude of 3.2 ${\text{km}}$. The less diluted cloud-core region subsumes the maximum value of the droplet number concentration and droplet size. Microphysics diagrams at other spatial scales are shown in the supplementary file (Fig. S1).

In what follows, we discuss the microphysical mixing diagram as in Bera et al., & Burnet and Brenguier at different length scales^21,67^. Mixing diagrams have been generated to ascertain the microphysical impact of mixing at distinct spatial scales, particularly over the finer scales where microscale vortices crucially modulate settling rates of small inertial cloud droplets. Figure 4 shows a mixing diagram (${N}_{d}-{r}^{3}$) at spatial scales 1 and 20 ${\text{cm}}$, where ${N}_{d}$ is droplet number density and $r$ is the droplet mean radius with its largest value ${r}_{a}$ in the cloud core.

Figure 4 points to a contrasting mixing characteristic in the central cloud core, core periphery, and the highly diluted fringe at two different length scales of 1 and 20 ${\text{cm}}$. Microphysics diagrams at other spatial scales are shown in Fig. S1, included in the supplementary file. It is observed that mixing is almost indistinguishable between the different cloudy zones at the smallest scale (1 ${\text{cm}}$), whereas a clear separation between the microphysical parameters is noted for the larger scale, i.e. 20 ${\text{cm}}$. It can be argued that at finer scales below a tenth of a centimetre, mixing is mostly determined by the small-scale local processes such as those mediated by rotating microscale vortices and other microphysical processes such as activation/condensation/evaporation modulated by the local water vapour field. For physical scales exceeding 10 ${\text{cm}}$, the mixing attributes within the cloud core and edge are uniquely distinguishable as seen from Fig. 4b. It is further noted that within the undiluted cloud core, the droplet radius gradually increases with an increase in the droplet number density. On the contrary, a decreasing trend is observed as one proceeds towards the cloud fringes where the droplet radius decreases with a decrease in the droplet count. This effect can be attributed to the mixing of dry ambient air which dilutes the droplet number density and pursues evaporation to restore the saturation balance.

It is also observed that several points in Fig. 4a share same ${r}^{3}/{r}_{a}^{3}$ value which is a characteristic feature of observational data as this is a Particle-by-Particle (PbP) measurement. Data points present within the same cloud volume may have similar mixing characteristics yielding the same ${r}^{3}/{r}_{a}^{3}$ values. The data is obtained by the cloud droplet probe (CDP-2) which measures the intensity of scattered light, which is then converted to droplet size (microns) using a calibration line into 30 size bins (between 2 and 50 microns). We have observed that although the intensity of scattered light varies continuously but after converting to sizes (in 30 bins), it has discrete values, and many droplets fall within the same size bin. Please note that this feature (dots sharing same ${r}^{3}/{r}_{a}^{3}$ values) is more prominent in small scale analysis (Fig. 4a); however, for larger scales (Fig. 4b), this feature vanishes due to averaging over many droplets. However, it is of further interest to investigate how droplets in these three distinct saturation regimes settle around eddy microscales of the order of a few millimetres embedded within the fabric of a turbulent cloud and collect other similar-sized droplets for effective cloud auto conversion.

**Figure 5:**
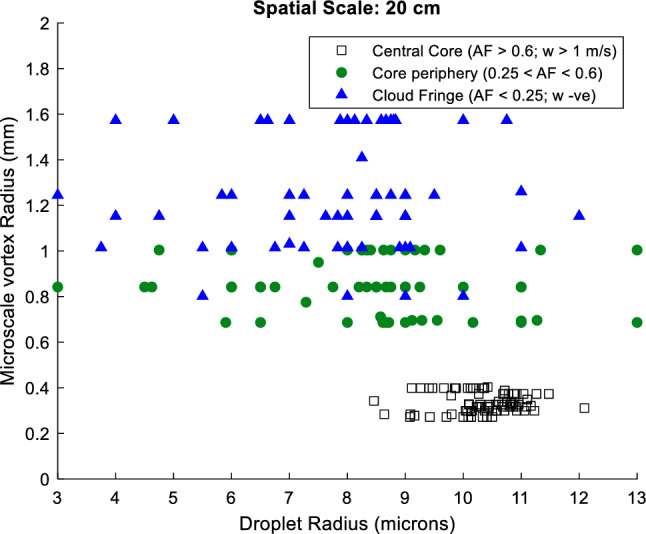
Microscale vortex radius versus droplet radius at a spatial scale of 20 ${\text{cm}}$. At spatial scales exceeding 5 ${\text{cm}}$, data points in the central core and the periphery are uniquely distinguishable. Also, note that droplets in the cloud core are centred around 10 $\mathrm{\mu m}$ and less scattered than the points in other cloud zones. These large droplets apprehend sub-millimetre-sized vortices with length scales between 0.2 and 0.4 ${\text{mm}}$.

Earlier studies have shown that the characteristic length scale of typical vortices within cumulus clouds is of the order of 1.0 ${\text{mm}}$^15,24,25,45^. This conforms with the present analysis as illustrated in Fig. 5. The characteristic length scale ($\eta$) of such eddies can be estimated from Eq. (7) (Davidson^68^) (refer to Table A1 for symbols):

**Table 1:** Mean and Standard deviation values for IPD, Flung Radii and ${I}_{r}$ for droplet radii 8, 10, 15 $\mathrm{\mu m}$. Note that the mean values of IPD and FR are equivalent and are of the same order in the central core.

Droplet radius ($\mathrm{\mu m}$)	Mean IPD/Standard deviation IPD ($\lambda ) {\text{mm}}$	Mean FR/Standard deviation IPD ($\lambda ) {\text{mm}}$	Mean ${I}_{r}$/Min ${I}_{r}$/Max ${I}_{r}$
8	11.96/12.57	42.04/7.818	16.19/0.69/212.07
10	10.54/9.89	27.56/4.39	8.90/0.46/140.42
15	10.18/8.28	11.59/1.78	2.49/0.34/6.70

7 $$\eta ={\left(\frac{{\nu }^{3}}{\epsilon }\right)}^\frac{1}{4}$$

Here, $\nu$ is the viscosity of air (${1.5\times 10}^{-5} {{\text{m}}}^{2}{{\text{s}}}^{-1}$) and $\epsilon$ the turbulent energy dissipation rate which is half the sum of the variances of the fluctuating velocity components $\frac{1}{2}({u}^{{\prime}2}+{v}^{{\prime}2}+{w}^{{\prime}2})$ obtained from real observations of three-dimensional wind velocity collocated at a high resolution of 20 ${\text{Hz}}$ by the AIMMS probe onboard the research flight and scaled with the time over which the energy within the cloud eddy dissipates. The maximum (minimum) turbulent energy dissipation rate ($\epsilon$) estimated from the wind velocity dataset in the central core, the core-periphery, and the cloud fringe was found to be 9000 (1200) ${{\text{cm}}}^{2}{{\text{s}}}^{-3}$, 150 (30) ${{\text{cm}}}^{2}{{\text{s}}}^{-3}$, and 80 (5) ${{\text{cm}}}^{2}{{\text{s}}}^{-3}$. Using the Eq. (7), the maximum (minimum) characteristic length scale ($\eta$) or the microscale vortex radii could then be estimated and were found to be 0.40 (0.24) ${\text{mm}}$, 1.00 (0.68) ${\text{mm}}$, 1.57 (0.80) ${\text{mm}}$. This is shown in Fig. 5.

It can be seen from Fig. 5 that microscale structures are smeared universally across all the cloud zones including the central core and the highly diluted cloud edges. The droplet-rich regions (with high values of liquid water content) in the central cloud core have been identified based on the adiabatic fraction (AF = $LWC/LW{C}_{ad}$) value of greater than 0.6 and vertical velocity ($w$) greater than 1 ${\text{m}}/{\text{s}}$. The core-periphery Zone 2 has an AF value between 0.25 and 0.6 and features positive vertical velocities (0 $<w<$ 1) and Zone 3, which is positioned along the cloud edges has been identified with AF less than 0.25 and negative updraught speeds ($w<0$). In the outermost Zone 3, one notices a preponderance of smaller droplets which may undergo complete or partial evaporation, depending on the available moisture mediated by the entrainment of cloud-free air. The variability observed in the droplet radius (blue triangular markers in Fig. 5) could be attributed to the differences in the available super-saturation. Meanwhile, there could also be some activation of condensation nuclei contributing to smaller droplets, which may interact with the MSVs. It can be further observed from Fig. 5 that these smaller-sized droplets are embedded within a fabric of microscale vortices with radii in the range of 1–2 ${\text{mm}}$. However, within these outer fringes, other large-scale processes such as entrainment dominate over fine-scale processes such as MSV-mediated mixing. It is also seen that droplets in the central core are positioned around 10 $\mathrm{\mu m}$ and are less scattered than those observed in the periphery (Fig. 5). These large droplets apprehend microscale eddies with radii between 0.2 and 0.4 ${\text{mm}}$ as also pointed out in an earlier study by Saitoh and Gotoh^69^. Conclusively, it is clear from Fig. 5 that droplets of all size classes in the range 3–13 $\mathrm{\mu m}$ interact with these millimetre-sized structures universally smeared across cloud saturation regimes, including the cloud core and the edges and this paper investigates some aspects of these interactions, particularly within the central core, which is shielded from extraneous effects, i.e. entrainment.

As these droplets settle past line vortices with a radius of $\sim {R}_{v}$ and circulation ~ $\Gamma$, the trajectories of droplets in a gravitational field, having fall speed in still fluid ~ ${V}_{TO}$ are determined by a balance between the settling process and the centrifugal effects of the particles’ inertia. This can be determined by the non-dimensional ‘particle Froude number’ ${F}_{p}$, which is the ratio of the stopping distance (${V}_{TO}{\tau }_{p})$ of the droplet to the characteristic radius ($\Gamma /{V}_{TO}$) of the trajectory of the droplet around the vortex^24^.

8 $${F}_{p}=\frac{{V}_{TO}{\tau }_{p}}{\Gamma /{{\text{V}}}_{{\text{TO}}}}$$

In the physical sense, when the effect of the particle inertia is very small (${F}_{p}\ll 1)$, it passes around the vortex and the net change in ${V}_{T}$ from its still-air fall velocity ${V}_{TO}$ is negligible. On the contrary, when the inertia is large enough, i.e. ${F}_{p}\sim 1$, the particles are centrifuged outwards so that the droplet settling rate is enhanced compared to its still-air settling rate. With a further increase in the particle size, i.e. inertia, the particles *crash* through these vortices and are not impacted by these turning microscale structures. Ghosh et al., found that the velocity amplification effect fades with increasing drop radii and becomes extremely small when droplet size approaches 40 $\mathrm{\mu m}$^25^. It is interesting to note that the droplet sizes observed during the CAIPEEX Phase IV experiment were in the range of 2–30 $\mathrm{\mu m}$ making it an ideal case setup to study the effect of circulating vortices on the droplet settling rates and the associated impacts on cloud microphysical processes.

Using the theory proposed by Dávila & Hunt, (2001), in this paper, we extend our calculations over a wide range of particle Froude number ${F}_{p}$ values^24^. The calculations sensitively depend on the droplet size, because the velocity amplification ratio ${V}_{e}/{V}_{TO}=(1-\alpha D)$, where ${V}_{e}$ is the droplet’s enhanced fall-velocity mediated by MSVs, $D$ is the average value of the ‘drift integral’ (which is a measure of the difference between the vertical settling distances with and without the vortex for particles starting at a fixed point and falling for a fixed period) for different values of ${F}_{p}$ and ${V}_{TO}$ appearing in the flow. Here, $\alpha$ is the effective volume fraction occupied by the vortices, so that $\alpha \sim ((\Gamma /{V}_{TO})/(\Delta {l}_{v}^{2}))$ and ${l}_{v}$ is the distance between the eddies. $D$ becomes more negative with decreasing values of ${V}_{TO}$, which varies as ${r}^{2}$ for small cloud droplets; thus, the ${V}_{TO}$ values become progressively smaller with smaller $r$ causing larger negative values of $D$ and larger amplification as should be.

For the observed value of the droplet radius as measured by the fast-response CDP probe, we first calculate the fall velocity ${V}_{TO}$, the particle Froude number ${F}_{p}$ and the volume fraction $\alpha$. Then, following the Dávila & Hunt (2001) theory^24^, the drift integral is evaluated, which can be written as:

9 $$D=\underset{-\infty }{\overset{\infty }{\int }}\Delta \eta \left({X}_{0}\right)\frac{d{X}_{0}}{\Gamma /{V}_{TO}}$$

**Figure 6:**
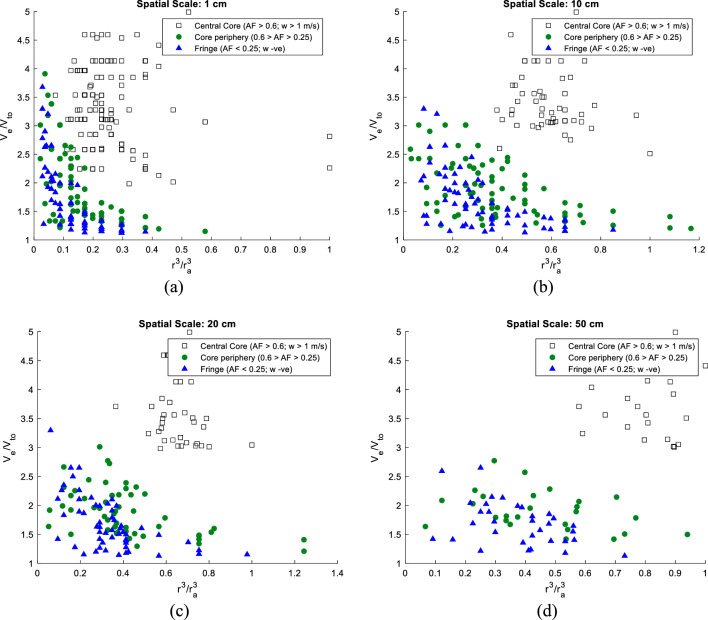
Droplet enhancement (${V}_{e}/{V}_{TO}$) versus ${r}^{3}/{r}_{a}^{3}$ for differing spatial scales, i.e. 1, 10, 20, 50 ${\text{cm}}$ in three cloud zones (less diluted cloud core $AF$ > 0.6; $w$ > 1 ${\text{m}}/{\text{s}}$, intermediately diluted cloud region $0.25<AF<0.6$ and highly-diluted cloud fringe $AF<$ 0.25; $w$ $-ve$ for a cloud penetration altitude of 3.2 ${\text{km}}$.

Here, $\Delta \eta \left({X}_{0}\right)$ is the dimensionless differential settling length with respect to settling in still fluid, a function of the initial horizontal position ${X}_{0}$, and $\Gamma /{V}_{TO}$ is the *Trajectory Radius* (${R}_{traj}$) or the *Flung Radius* as mentioned earlier. From an estimation of the drift integral, which was always negative over the sampled droplet datasets, it was possible to quantify the extent of velocity enhancements for the droplets within the cloud. In what follows, the velocity amplification diagrams are produced at different spatial scales correlating droplet velocity enhancement (${V}_{e}/{V}_{TO}$) with ${r}^{3}/{r}_{a}^{3}$. It can be seen from Fig. 6 that droplet settling rates for very small droplets positioned within the central core are up to five times higher than their still-air settling speeds. However, microscale processes only have a limited impact in the outermost zones where other large-scale processes like entrainment take over and so the settling rates of droplets in the crucial size range of 10–18 $\mathrm{\mu m}$ (${r}^{3}/{r}_{a}^{3}$ ~ 0.7) are almost equivalent to their still-air settling speeds. In the innermost cloud zone, which is greatly shielded by large-scale mixing, droplets subsume near-adiabatic sizes and undergo enhanced settling between two to five times their still-air fall speeds. Within this region, other extraneous effects are subdued so droplet growth post-condensation may be attributed to droplet-vortex interactions. It can be further noted from Fig. 6 that at spatial scales equal to or exceeding 10 ${\text{cm}}$, droplet settling patterns in the core and edge are uniquely distinguishable.

**Figure 7:**
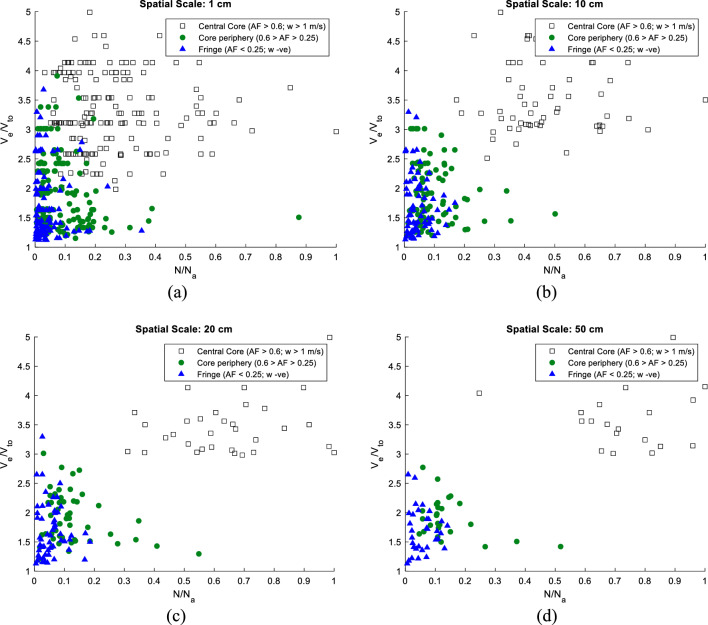
Droplet velocity enhancement (${V}_{e}/{V}_{TO}$) versus $N-{N}_{a}$ for differing spatial scales, i.e. 1, 10, 20, 50 ${\text{cm}}$ in three cloud zones (less diluted cloud core $AF$ > 0.6; $w$ > 1 ${\text{m}}/{\text{s}}$, intermediately diluted cloud region $0.25<AF<0.6$ and highly-diluted cloud fringe $AF<$ 0.25; $w$ $-ve$ for a cloud penetration altitude of 3.2 ${\text{km}}$.

Because of the centrifugal inertial force of the rotating microstructures, particles released inside the vortex gradually drift outwards. With a handle on the overall fluid velocity and the characteristic length scales of vortices within the cloud zones, it is possible to estimate vortical strengths ($\Gamma \sim \eta \times U$) within these zones. It is estimated that the vortical strength inside the central core is higher (${\Gamma }_{core} \sim$ 2.31–7.52 $\times {10}^{-4}{\mathrm{ m}}^{2}{{\text{s}}}^{-1}$) than in the peripheral regions (${\Gamma }_{per} \sim$ 0.53–1.2 $\times {10}^{-4}{\mathrm{ m}}^{2}{{\text{s}}}^{-1}$) indicating that MSV effects are higher in the innermost zone. For instance, droplets in the similar size range of 10–18 $\mathrm{\mu m}$ (${r}^{3}/{r}_{a}^{3}$ ~ 0.7) have higher flinging rates in the innermost zone than in the core-periphery. It is further observed from Figs. 6 and 7 that the droplet fall-velocity enhancements progressively wane with increasing droplet sizes as pointed out in earlier studies by Ghosh et al., and Dávila & Hunt^24,25^.

From Fig. 7, one notices a higher number density of large droplets in the less-diluted cloud core than at the edges. Since the droplets in the innermost zone are more in number and are homogeneously distributed around large sizes, i.e. ${r}^{3}/{r}_{a}^{3}\to 0.7$ than in other pockets (as seen from Fig. 6), there is a greater propensity for self-collection of these similarly sized droplets which have greater settling speeds than in still air. With a wide range of drop sizes greatly impacted by MSVs, the flinging action of vortices contributes largely to the observed homogeneous Poisson statistics in the Bera et al., study^21^. The MSV effect promotes greater mixing so that even when an assortment of cloud droplets is homogeneously distributed, there can be rapid droplet growth causing spectral broadening.

This may well be a possible explanation for the first raindrop formation in slightly diluted volumes in the upper reaches of the cloud where the cloud liquid water is also at its maximum. By definition, the self-collection of similarly-sized larger droplets with larger settling speeds in the core will only aid further collisions supporting the argument posed by earlier studies that the first raindrops form in the adiabatic (or nearly adiabatic) volumes^5^. These conditions may also aid in the formation of super adiabatic droplets through effective collision and coalescence^70,71^.

**Figure 8:**
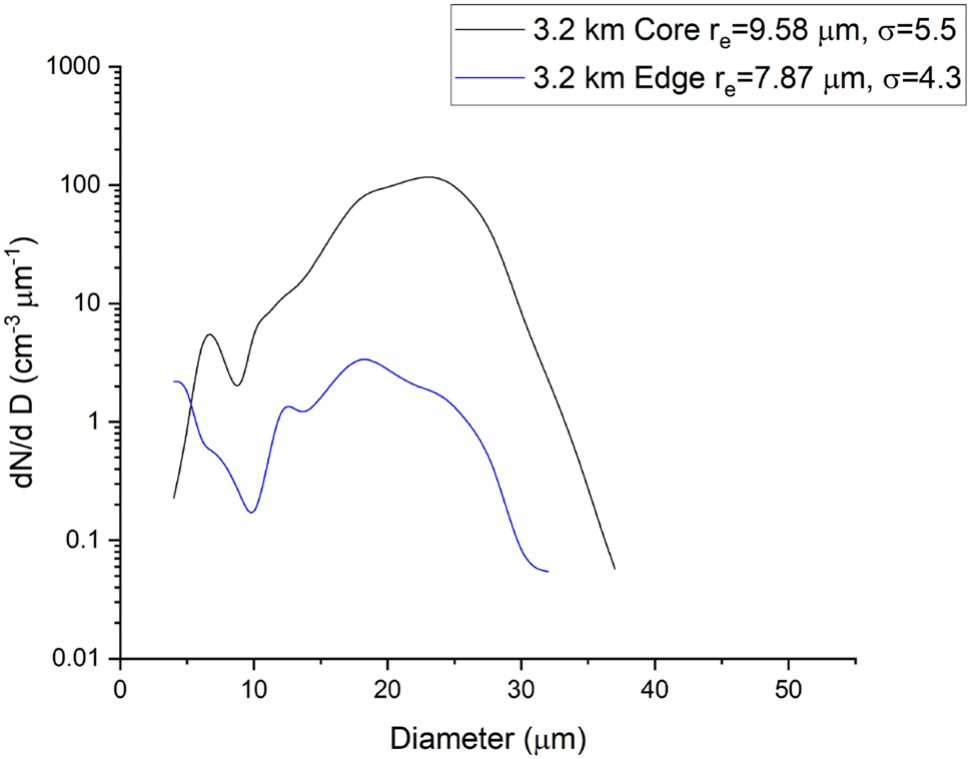
Droplet size spectra in cloud core and edge regions corresponding to the entire flight pass at 3.2 ${\text{km}}$. Values of mean radius (${r}_{e}$; $\mathrm{\mu m}$), spectral width ($\sigma$; $\mathrm{\mu m}$) corresponding to each spectrum are also provided in the figure. Note a 30% higher $\sigma$ in the cloud core compared to the value observed in the cloud fringes implying spectral broadening mediated by the flinging action of microscale vortices.

**Figure 9:**
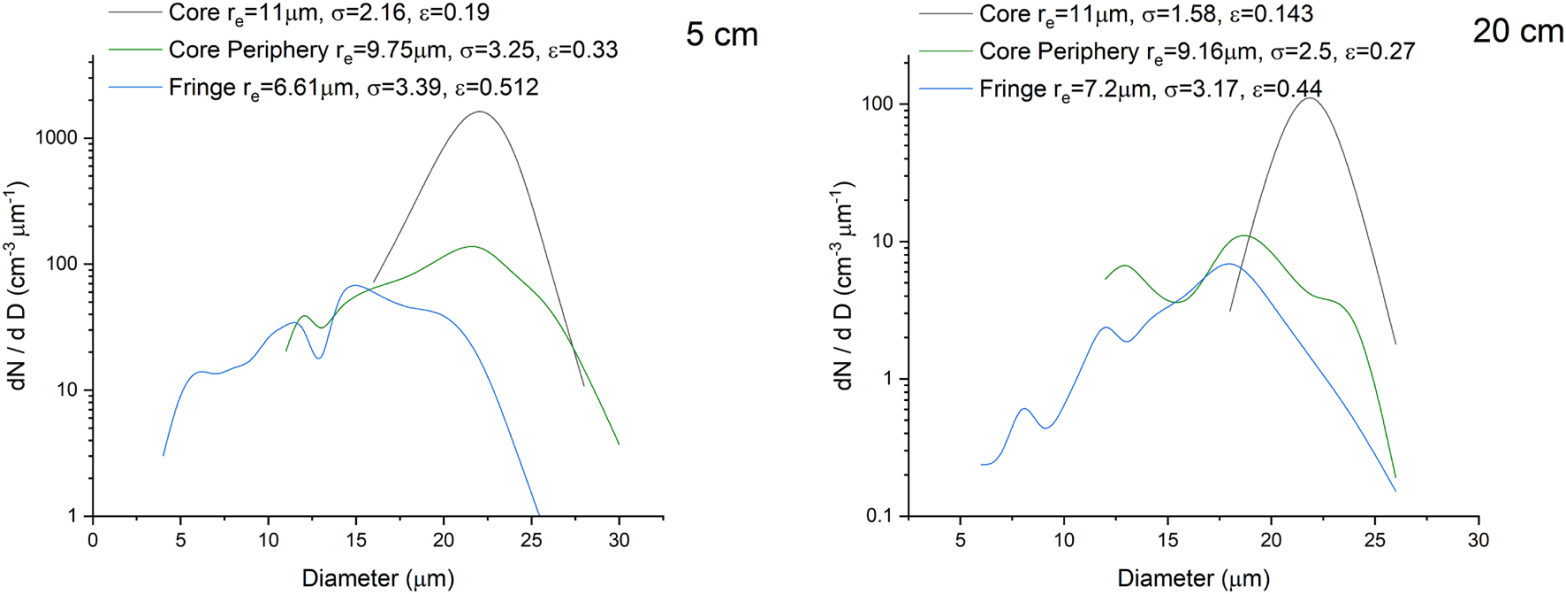
Droplet size spectra in cloud core, core periphery and fringes corresponding to the flight pass at 3.2 ${\text{km}}$ at different spatial scales. Values of mean radius (${r}_{e}$; $\mathrm{\mu m}$), spectral width ($\sigma$; $\mathrm{\mu m}$) and relative dispersion ($\epsilon$) corresponding to each spectrum are also provided in the figure. Note the shifting of the spectra to higher sizes in the core and its peripheries compared to the spectrum in the cloud fringe.

It is earlier premised that cloud droplets in a critical size range are shielded from the effects of entrainment in the core. It is here that one would expect to observe droplet spectral broadening without external influences. In Figs. 8 and 9, we have shown the overall, and spatial scale-wise drop size distributions in the core, in the peripheries and in the edges, where broadening of spectra to larger sizes is visible in the core and its peripheries and is larger here compared to the spectrum at the edges. The second distinct mode is due to broadening of the spectra, and this is more pronounced in the core regions. In Fig. 8, the droplet-rich regions (with high values of liquid water content) in the central cloud core have been identified based on the adiabatic fraction value of greater than 0.6 and vertical velocity (w) greater than 1 m/s, whereas the edge has a lower AF value with negative updraught speeds (w < 0). One notices a 30% higher σ in the cloud core compared to the value observed in the cloud fringes implying spectral broadening mediated by the flinging action of microscale vortices.

Scale-wise drop size distribution is shown in three cloud regions identified as core and its periphery and the cloud fringe. The core-periphery has an AF value between 0.25 and 0.6 and features positive vertical velocities (0 $<w<$ 1). Broadening of the cloud spectra over larger sizes is again visible in the cloud core and its peripheries combined compared to the spectrum in the cloud edge. Relative dispersion ($\epsilon$) within different cloud zones is also supplied in the figure legends wherein a lower dispersion is noted in the central region, which is now discussed.

**Figure 10:**
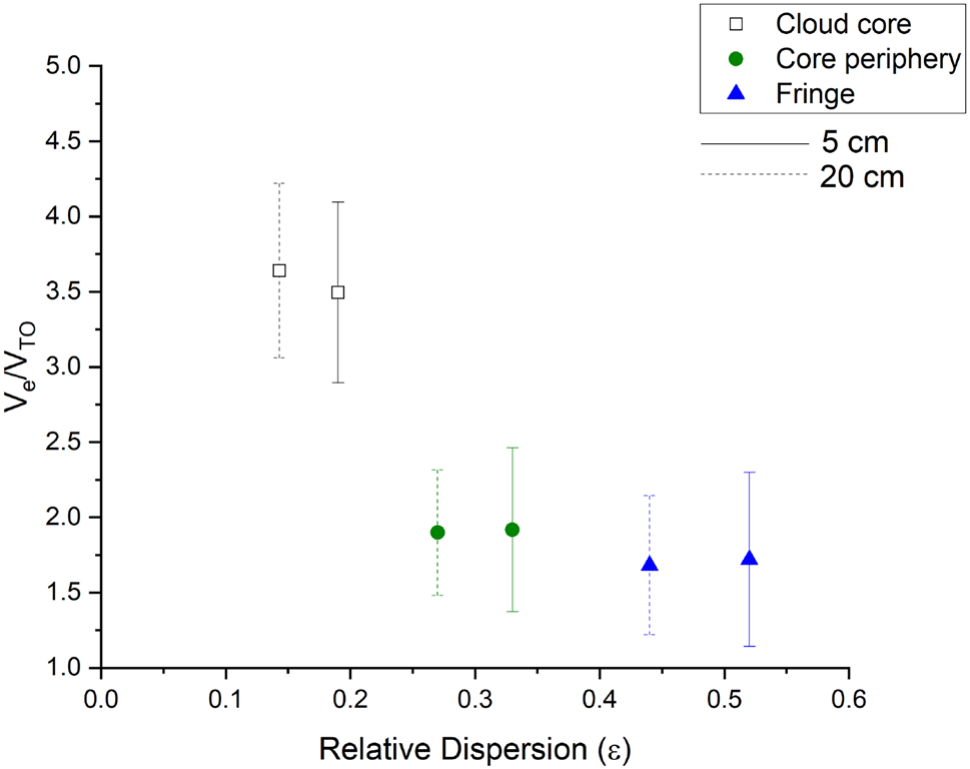
Droplet velocity enhancement (${V}_{e}/{V}_{TO}$) versus $\epsilon$ for differing spatial scales, i.e. 5 and 20 ${\text{cm}}$ in three cloud zones. Note how collision and coalescence between droplets aided by the flinging action of microscale vortices in the cloud-core (black colour) yield droplets centred around larger values thereby lowering relative dispersion in the cloud-core.

Figure 10 relates velocity amplification to relative dispersion in the cloud zones. It is noted that because microscale vortices are flinging droplets of all sizes including ones within the critical range (6–18 $\mathrm{\mu m}$) and covering a wide range of dissimilar sizes for efficient coalescence as observed, the settling droplets now traverse longer path lengths allowing for them to be captured and collected. This results in the production of larger size droplets which grow concomitantly and without the extraneous effects in the core, the relative dispersion is lower in the central region.

In summary, these CAIPEEX observations indicate that the conditions most favourable to rain formation happen in the core for droplets in the range 10–18 $\mathrm{\mu m}$ which are amplified by up to 4 times causing spectral broadening in the core regions where the flung radii are also equivalent to IPD (Fig. 12). In the next sections, we show the effect of these processes in large scale models.

**Figure 11:**
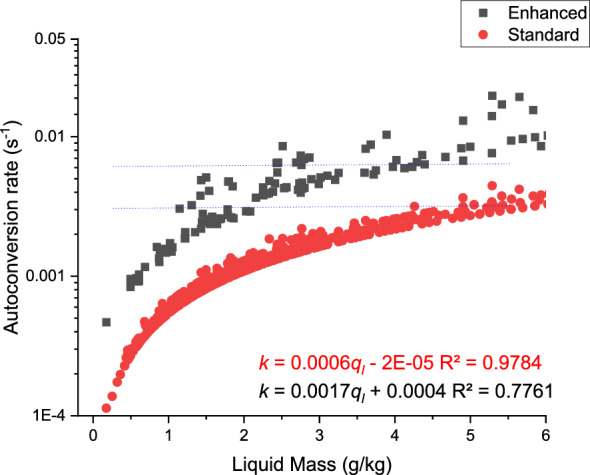
Cloud auto-conversion rates (${k}_{1}$) (${{\text{s}}}^{-1}$) versus cloud liquid mass (${\text{g}}/{\text{kg}}$) for the two cases, i.e. Standard and Enhanced test cases.

Figure 11 shows the standard (${k}_{1}$) and modified autoconversion rates (${k}_{1}{\prime}$) for different values of the cloud liquid water content.

As expected, the autoconversion rate increases with increasing values of the cloud liquid mass. From the preceding Figs. 6 and 7, it was noted that the droplets in the central core mainly subsumed sizes around their adiabatic values of around 10–18 $\mathrm{\mu m}$. With a handle on droplet sizes and the associated numbers in the innermost cloud zone, it was possible to estimate the total liquid mass of droplets in this region, which was found to be in the range of 2–6 ${\text{g}}/{\text{kg}}$ as can be seen from Fig. 11. In fact, for a maximum value of the liquid water content of 6 ${\text{g}}/{\text{kg}}$, the autoconversion rates ${k}_{1}$ and ${k}_{1}{\prime}$ are estimated to be 0.003 and 0.006 ${{\text{s}}}^{-1}$, respectively for the two cases.

It is observed that processes related to self-collection and auto-conversion of cloud water to rainwater are mediated strongly by microscale vortices in the central core, thus governing droplet groupings in the cloud core. In what follows, new observations are presented on how droplet separation is impacted by the flinging action of microscale vortices in turbulent clouds over a select radii range and how they also vary over cloud cores and along the peripheral edges. It is premised that this mechanism initiates droplet separation within a cloud mass soon after condensational growth largely in the cloud core and operates until their radii exceed 20–30 $\mathrm{\mu m}$ when this effect fades rapidly.

**Figure 12:**
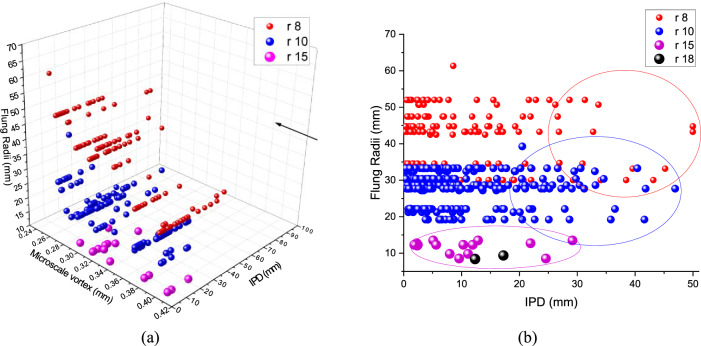
Droplet flung radii (${\text{mm}}$) versus droplet interparticle distances (${\text{mm}}$) in the cloud’s central core for smaller and larger droplet sizes ($r$: 8, 10, 15 and 18 $\mathrm{\mu m}$). Note that the inter-particle distance of two consecutive droplets varies from 0.2 to 50 ${\text{mm}}$ in the central core. Lighter particles, i.e. $r$: 8 $\mathrm{\mu m}$ can be seen levitating higher up over larger values of the flung radii approaching 60 ${\text{mm}}$ for which even IPDs extend up to 50 ${\text{mm}}$. Heavy droplets with larger radii, i.e. $r$: 18 $\mathrm{\mu m}$ are positioned over a smaller flung radii range spanning up to 10 ${\text{mm}}$ and their IPDs are also restricted up to 18 ${\text{mm}}$. Note that regions where the IPDs are equivalent to the Flung Radii are marked in ellipses.

The observational datasets were used to correlate droplet interparticle distances with estimated droplet *trajectory radii* (we call this the *flung radius*) as they approached line vortices. This is perhaps the first mission to record observations on the droplet inter-particle distances which is a useful quantity to study droplet dynamics in a turbulent medium characterised by moving line vortices. In particular, we have analysed some properties of the droplet trajectories and have correlated them with droplet arrival distances. Figure 12 shows a relationship between the Droplet flung radii (${\text{mm}}$) or the trajectory radii (${R}_{traj}$) versus droplet interparticle distance (IPD) for the smaller and the larger droplet sizes observed within the cloud’s central core where other large-scale processes such as entrainment are minimal.

It can be seen that the flung radii in the cloud-core scale well with the measured values of the droplet interparticle distances indicating that microscale vortices impact droplet trajectories within the innermost cloud region modulating their inter-particle distances. The correlation between the two quantities wanes out as one proceeds towards the unshielded cloud fringes where other large-scale effects, such as entrainment of dry air dominate fine-scale mixing processes. For these CAIPEEX in-core droplets, the observed level of turbulent intensities is between moderate to high (0.003–6.35 ${{\text{m}}}^{2}{{\text{s}}}^{-2}$) and compares well with earlier reports on in-cloud turbulent intensities^69^. For the observed values of $\epsilon$ for the CAIPEEX in-cloud central core, the vortical radii range between 0.2 and 0.4 ${\text{mm}}$, i.e. submillimetre microscale eddies. It is clear that as discussed earlier droplets with radii between 6 and18 $\mathrm{\mu m}$ have the maximum velocity amplification indicating that they settle faster as they negotiate these varying eddy length scales. In the Bera et al. study, to date the most definitive observational analysis of IPD, it was shown that IPD varies significantly from a few tens of ${\text{mm}}$ to 50 ${\text{mm}}$ in the central core of the cloud^21^. It is premised that the shielded core region with IPD variability ranging between 0.2 and 50 ${\text{mm}}$ provides the best test example to ascertain for the first time how processes that are free of extraneous effects, i.e. entrainment can provide the most suitable platform to provide a correlation between the flinging action of these rotating rankine vortices with the observed IPDs. A varying assortment of droplet sizes interacts with varied eddy sizes, and one would expect the largest droplets would be least affected by rotating eddies and the smallest would be the most affected by these rotating structures. We can see from Fig. 12 that for the droplets with radii 8 $\mathrm{\mu m}$, the spread between the IPD varies between 0.2 and 50 ${\text{mm}}$, and the flung radii are also the largest going up to 60 mm. For the largest droplets, the IPD is confined to only 30 ${\text{mm}}$ with flung radii also confined to the smaller values, i.e. within 15 ${\text{mm}}$ suggesting that these very large droplets are affected only to a small percentage by the flinging action of rotating eddies. It is quite clear that the homogenous distribution of droplets in the core as shown by Bera et al. can be attributed to the microscale mixing mediated by the flinging action of the rotating vortices centrifuging differently sized droplets to different flung radii and contributing proportionately to the IPD variability^21^. Only when the droplet size increases beyond 20 $\mathrm{\mu m}$, does the MSV effect fade and other large-scale processes, i.e. entrainment take over. The latter, as expected, is greatest at the edges for the larger drops.

Small-scale microscale mixing is felt the maximum in the cloud core when the droplets are shielded from outside air. It must be emphasised that a) the strength of microscale vortices is related to the rescaled Stokes number implying that when the droplets are very small ($r\cong$ 1 $\mathrm{\mu m}$), they exactly follow the fluid flow as they behave as inertia-less particles^25^. When they are very large ($r>$ 20 $\mathrm{\mu m}$), the effect starts to fade because their inertia is large enough that the presence of these rotating eddies is not noticed, and the droplets simply crash through them. In the intermediate critical range, the effect is most pronounced as is discussed. These small processes are seen to have a modulating effect over a limited but nevertheless important radius range, particularly in the core where other effects like entrainment are minimal. Lateral entrainment in cumulus clouds is more effective in cloud edges whereas core is relatively less impacted. Also, entrainment dynamics starts at larger scales (100–200 ${\text{m}}$) and then propagates to smaller scale with decreasing kinetic energy^72–75^. In this study, our focus is on small scale cloud processes (millimetre scales) in the core and its peripheries, where entrainment effects are at their minimum. This line of reasoning prompts us to formulate a new number ${I}_{r}=\frac{FR}{\uplambda }$. When the ${I}_{r}\ge 1$, then the MSV effect is strongest. This is shown In Table 1.

These results are general to all cumulus/convective clouds irrespective of geographical location. Microscale vortices exist in all cloud types. Further, the strength of these vortices may have little dependence on the available TKE within clouds^25^. For example, continental cumulus clouds are generally more turbulent due to higher droplet concentrations implying lower IPDs as observed^21^. In contrast, IPDs in marine clouds may be larger due to lower droplet number concentration and in both cases, droplet grouping within clouds is impacted by the presence of microscale eddies.

With a clear illustration of how MSV affects droplet organisation within clouds, we now show the equilibrium rain-drop size distributions accounting for the enhanced velocities of the collected drops $(r)$ and compare the modelled drizzle spectra with CAIPEEX observations. Earlier studies by Baker^46^ and Austin et al.^76^ derived the equilibrium raindrop size distributions as a function of height $(z)$ for a spreading plume of raindrops 46, 75]. The former study completely ignored the turbulent fluctuations within the cloudy mass, whilst the latter study accounted for turbulence-mediated air velocity fluctuations. Additionally, Austin et al. also included the settling rates of the large collector drops $\left(R\right)$ sedimenting past a stationary group of cloud droplets^76^. As noted earlier, cloud droplet speeds can be significantly higher and must be incorporated. A later study by Ghosh and Jonas modified the raindrop size distribution to incorporate the amplified velocity of the collected droplet $(r)$ in the collection times $({\tau }_{c}^{*})$^45^.

The probability of spotting a raindrop at a height $z$ which originated within a cloud layer at a height $z{\prime}$ at time $t$ is ${f}_{r}\left(z,{z}{\prime},t\right)$. Here, ${f}_{r}$ is governed by the advection–diffusion equation given below.

10 $$\frac{{\partial f_{r} \left( {z,z^{\prime } ,t} \right)}}{\partial t} + U\left( t \right)\frac{{\partial f_{r} \left( {z,z^{\prime } ,t} \right)}}{\partial z} = D_{zz} \frac{{\partial^{2} f_{r} \left( {z,z^{\prime } ,t} \right)}}{{\partial z^{2} }}$$

Here, $U\left(t\right)=w-|V(R\left(t\right)-V(r)|$, $w$ the velocity fluctuation, $V(R)$ the velocity of the large collector drops $(R)$, and $V(r)$ the amplified velocity of the small cloud droplets. At the onset, the fraction of raindrops emanating from a point burst at height $z{\prime}$ is given by an impulse function ${f}_{r}\left(z,{z}{\prime},t\right)=\delta (z-{z}{\prime})$. Further, the arrival rate of the raindrops at height $z$ is given by: $S(z,{z}{\prime},t)dz{\prime}$ $({m}^{3}/s)$ = $F\left(z,{z}{\prime},t\right)dz{\prime}Q$, here $Q$ is the magnitude of the raindrop source linked to the cloud auto-conversion rate (${k}_{1} or {k}_{1}{\prime})$ $Q=\frac{{k}_{1}}{4\pi {R}_{0}^{3}{\rho }_{l}/3}$ and is estimated to be 2 $\times {10}^{-6}$ ${{\text{kgm}}}^{-3}$ and 6 $\times {10}^{-7}$ $kg{m}^{-3}$ for the two cases. A Fourier transform of Eq. (10) yields the arrival rate of the raindrops to be:

11 $$S\left( {z,z^{\prime},t} \right) = \frac{Q}{{\sqrt {2\pi } \sigma_{z} }}\mathop \smallint \limits_{0}^{h} \exp \left[ {\frac{{ - \left\{ {z - z^{\prime} - \left\{ { - JR_{o} \left( {\left( {\frac{t}{{\tau_{c}^{*} }}} \right) - 1} \right) - k^{\prime}t \times r^{{2 + w_{2} }} } \right\}} \right\}^{2} }}{{2\sigma_{z}^{2} }}} \right]dz^{\prime}$$

The above equation can be integrated over a cloud thickness $h$ to yield the equilibrium raindrop size distribution given by:

12 $$\begin{aligned} \eta \left( {z,R} \right)dR = & \frac{{Q\tau_{c}^{*} }}{2R}\left[ {{\text{erf}}\left( {\frac{{z - \left\{ { - JR_{o} \tau_{c}^{*} \left( {exp\left( {\frac{t}{{\tau_{c}^{*} }}} \right) - 1} \right) - k^{\prime}t \times r^{{2 + w_{2} }} } \right\}}}{{\sqrt 2 \sigma_{z} }}} \right)} \right. \\ & \;\left. { - {\text{erf}}\left( {\frac{{z - \left\{ { - JR_{o} \tau_{c}^{*} \left( {exp\left( {\frac{t}{{\tau_{c}^{*} }}} \right) - 1} \right) - k^{\prime}t \times r^{{2 + w_{2} }} } \right\} - h}}{{\sqrt 2 \sigma_{z} }}} \right)} \right] \\ \end{aligned}$$

**Figure 13:**
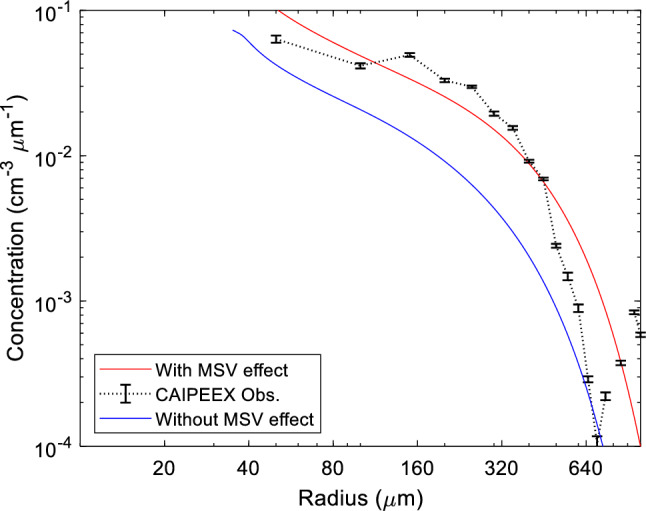
Equilibrium Raindrop size distribution at 100 ${\text{m}}$ from the cloud top with (red line plot) and without (blue line plot) the MSV effect. Note that the CAIPEEX in-situ rain observations are shown as error bars stitched together (black dotted line) and agree well with the case when MSV effects are accounted for in the modelled distribution.

Here, ${R}_{o}$ is the initial raindrop radius, ${k}{\prime}={k}_{0}{w}_{1}$, where ${w}_{1}$ (20) and ${w}_{2}$ ($-$ 0.168) are weighted constants accounting for the cloud droplet amplification effect, $t$ is given by ${\tau }_{c}^{*}{\text{ln}}(R/{R}_{0})$, $h$ is the cloud depth, ${\sigma }_{z}$ is the plume standard deviation as given in Ghosh & Jonas and Austin et al.^45,76^. By substituting ${w}_{1}\to 1$, ${w}_{2}\to 0$, one obtains results pertaining to the case when the cloud droplets settle in still air. The equilibrium rain-drop size distribution is shown below in Fig. 13:

Note that the CAIPEEX in-situ rain observations shown in black dotted line agree well with the case when MSV effects are accounted for in the modelled distribution. The collection times (${\tau }_{c}$) for the two cases is estimated to be 538 ${\text{s}}$ (with the MSV effect) and 766 ${\text{s}}$ (without the MSV effect)- a ~ 30% decrease in the collection times indicating that larger *collector* droplets are able to quickly collect smaller *cloud* droplets when their hastened speeds mediated by line vortices are accounted for in the calculations. In the next section, we show how the rain onset, cloud and rain mixing ratios, and overall cloud morphology are impacted when these effects are accounted for in the WRF model.

### Results from an LES case study

**Figure 14:**
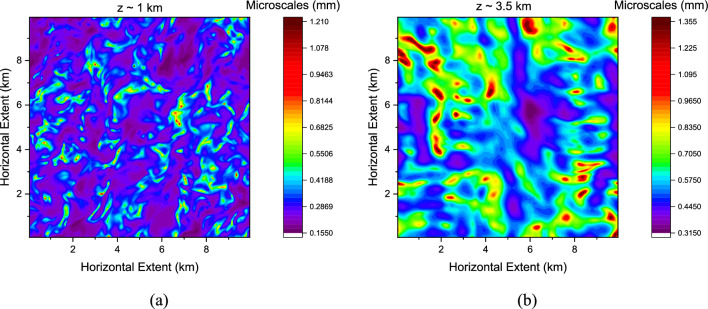
Two-dimensional layer snapshot of microscale eddies (0.2–1.35 ${\text{mm}}$) just below the (**a**) cloud base at $z\sim$ 1 ${\text{km}}$ and (**b**) towards the cloud top at $z\sim$ 3.5 ${\text{km}}$.

The case study under consideration is that of a convective cloud event where buoyancy-driven convection was primarily responsible for the turbulent transport of moisture. The modelled Turbulent Kinetic Energy (TKE ${{\text{m}}}^{2}{{\text{s}}}^{-2}$) profile is characterised by a peak with a local maximum in the sub-cloud region (1000 ${\text{m}}$) followed by a minimum towards the cloud-top (between 4 and 5 ${\text{km}}$) close to the inversion layer. This observation is typical of many convective boundary layers^77,78^. Moreover, a high ${w}^{{\prime}2}$ of ~ 2 ${{\text{m}}}^{2}{{\text{s}}}^{-2}$ in the sub-cloud region indicates the evolution of updrafts enabling the growth of clouds within an unstable atmospheric boundary layer. This has a direct bearing on the extent of liquid water generation modulated by the prevailing supersaturation. The modelled turbulent kinetic energy (TKE) varied between 2.68–5.9 ${{\text{m}}}^{2}{{\text{s}}}^{-2}$, 0.297–2.96 ${{\text{m}}}^{2}{{\text{s}}}^{-2}$ midway through the cloud and at the upper reaches, respectively. With the estimated values for $\epsilon$, it was possible to generate a two-dimensional coverage of microscale eddies as is shown in Fig. 14.

One notices that the small-scale turbulence organises itself in filament-like, cylindrical structures in the form of micro-scale tubules. The above snapshot of modelled eddy coverage illustrates an assortment of eddy length scales between $\sim$ 0.2 and 1.35 ${\text{mm}}$ and the modelled range of values agree well with the estimated values of length scales from observations (Fig. 5). Having described patterns of MSV coverage for the case study vis` a vis` microphysical modulation of cloud and rainwater, we now illustrate the modelled cloud vertical profiles and morphologies for the case study under consideration.

**Figure 15:**
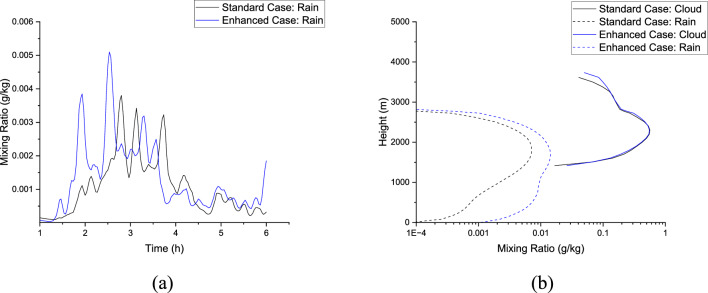
(**a**) Temporal evolution of domain-averaged rain mixing ratio for the Standard and Enhanced cases (**b**) Vertical profiles of layer-averaged cloud and rain mixing ratios for the Standard and Enhanced cases at $t$: 2.53 ${\text{h}}$.

Figure 15 shows the domain-averaged modelled rainwater amounts for the ‘enhanced microphysics case’ along with the results from the ‘standard’ baseline test case. One notices that rain amounts are underpredicted significantly in the standard case. Additionally, there is an early commencement of rainfall in the ‘enhanced’ case where fall-velocity amplification effects have been accounted for in the model run compared to the standard baseline case with still-air fall velocities.

**Figure 16:**
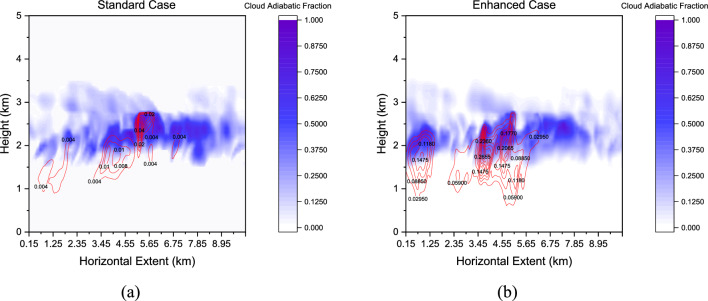
Instantaneous snapshots of modelled cloud morphology (Adiabatic Fraction) obtained from the LES model at $t$: 2.53 ${\text{h}}$ (**a**) Standard case (**b**) Enhanced case. Note that the rain mixing ratios are shown in red contours.

Whilst the layer-averaged vertical profiles shown in Fig. 15b also yield significant differences in the cloud and rain amounts, it is also necessary to explain variations in the cloud morphologies shown in Fig. 16. Figure 16 illustrates the cloud morphology for the two cases, i.e. ‘Standard’ and ‘Enhanced’ under consideration at $t$: 2.53 ${\text{h}}$ when a clear distinction was noted in the modelled rainfall (see Fig. 15a). A clear cloud base at 1.5 ${\text{km}}$ is observed with differentiated cloud and rain amounts in both cases.

Not only are the overall positioning of the cloud decks different, but the cloud morphological developments are also very different. Note the presence of red contour bands with intense precipitation activity in the ‘Enhanced’ case- the maximum values of rain mixing ratios in the two cases were found to be 0.26 ${\text{g}}/{\text{kg}}$ and 0.04 ${\text{g}}/{\text{kg}}$, respectively for the ‘enhanced’ and ‘baseline’ cases.

## Conclusions

Global warming-mediated weather extremes is likely to become the new norm in the coming decades. Short-duration extreme precipitation events cause local flooding at catastrophic levels requiring the evacuation of hundreds of thousands of people, whilst devastating droughts impact livelihoods and food production at the other extreme^79^. These disturbing trends are linked to the distribution of water resources across continents. In a monsoon-driven economy, as in India, one must re-examine the microphysics of clouds and precipitation. Engineering solutions can emerge from new conceptual advancements garnered from in situ observational studies and high-resolution modelling studies. This study builds up on the CAIPEEX observational data set that provided for the first-time cloud microphysical observations from a Cloud Droplet Probe (CDP-2 with K-tips) to record cloud attributes (spanning a diameter range of 2–50 µm) at 25 ${\text{Hz}}$. This unique repository of data analysed is consistent with the level of sophistication necessary for the understanding of rain formation processes. The present study shows that the flinging action of rotating Rankine-type vortex tubes impacts differently sized cloud droplets differently contributing to the droplet Inter Particle Distances or the IPDs. The IPD variations are different over cloud cores and the entrainment-dominated cloud edges. The IPD is a starting point for tracing droplet trajectories around these microscale vortices—when droplets with differing radii descend and encounter a rotating vortex, they can come closer to each other favouring collision coalescence. Not only do they come closer to each other, over a critical radii range of (6–18 $\mathrm{\mu m}$), they settle faster with amplified velocities affecting cloud water amounts converting to rain amounts in warm processes. The observations show that the IPD-Flung radii correlation is the strongest in the cloud cores (Adiabatic Fractions > 0.6 and vertical updraught $w$ > 1 ${\text{m}}/{\text{s}}$) and for the crucial droplet size range, the IPD values are equivalent to the flung radii. The flinging action of the vortices is so pervasive because, at these smallest scales, they scale with the viscosity and not the TKE dissipation rate. This IPD-MSV linkage is explored within the remit of the WRF-LES model for this case study. It is shown for the first time that the modelled equilibrium raindrop size distribution matches quite well with the observed raindrop spectra when MSV-mediated amplified fall velocities are used. It is suggested that models use a better power law for describing cloud droplet fall velocities when droplet radii are less than 20 $\mathrm{\mu m}$. Only thereafter, the microphysical formulation can revert to the still air values. The collection time (${\tau }_{c}$) in the ‘enhanced’ microphysics case is hastened by 30% indicating that larger collector droplets can quickly collect other smaller cloud droplets to yield higher rain amounts as observed from both experimental and modelling analysis.

## Supplementary Information

Supplementary Information.

## Data Availability

All data generated or analysed during this study are included in this published article [and its supplementary information files].
