# Supplementary material for: Turbulence-induced droplet grouping and augmented rain formation in cumulus clouds
Source: Sci Rep. 2024 May 4;14:10298. doi: 10.1038/s41598-024-61036-z (PMC11636904; doi:10.1038/s41598-024-61036-z)
Supplement: Supplementary file 1 — Supplementary Information. [file 41598_2024_61036_MOESM1_ESM.docx]

(a) (b)


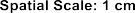

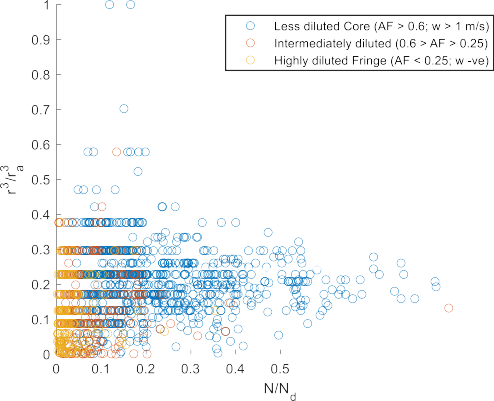

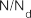

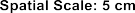

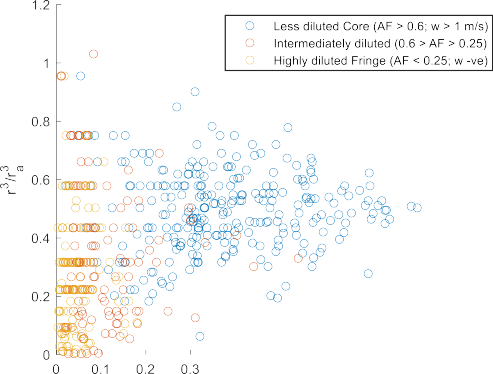

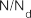

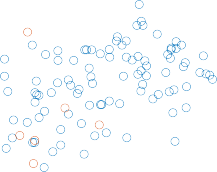

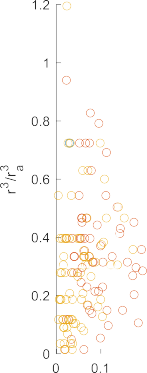

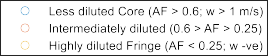


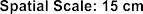


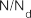

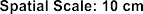

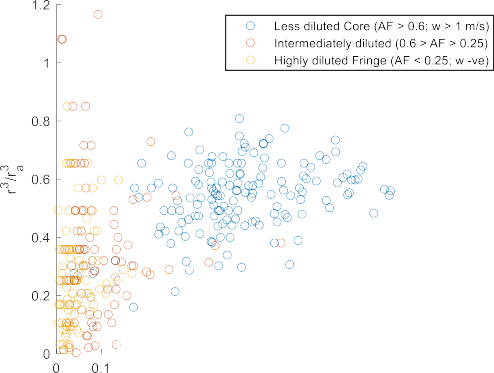


(c) (d)


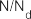

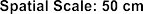

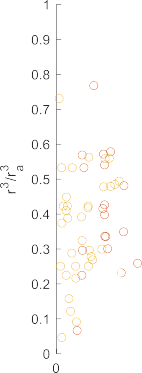

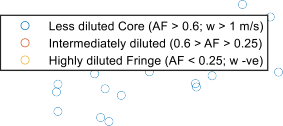


(e)

**Figure S1:** Microphysics diagram at different spatial scales (1, 5, 10, 15, 50 𝑐𝑚). The less diluted cloud-core region subsumes the maximum value of the droplet number concentration and droplet size.

Table A1: Symbols and Abbreviations

| $AF$ | Adiabatic Fraction |
| --- | --- |
| $S$ | Arrival rate of raindrops at height $z$ |
| $k_{1}$ | Auto-conversion rate ($s^{-1}$) |
| $a$ | Auto-conversion threshold ($gm^{-3}$) |
| $\eta$ | Characteristic length scale of microscale eddies ($mm$) |
| $r_{a}$ | Cloud droplet’s adiabatic size |
| $r$ | Cloud droplet radius ($\mu m$) |
| $m$ | Cloud mass ($gm^{-3}$) |
| $h$ | Cloud thickness |
| $\tau_{c}$ | Collection time |
| $\rho_{l}$ | Density of liquid ($kgm^{-3}$) |
| $\Delta\eta\left( X_{0} \right)$ | Dimensionless differential settling length with respect to settling in still fluid |
| $V_{e}$ | Droplet enhanced fall-speed mediated by microscale vortices ($cms^{-1}$) |
| $V_{TO}$ | Droplet fall-speed in still-air ($cms^{-1}$) |
| $N_{d}$ | Droplet Number density ($cm^{-3}$) |
| $\tau_{p}$ | Droplet phase-relaxation time ($s$) |
| $\alpha$ | Effective volume fraction occupied by the vortices |
| $\eta$ | Equilibrium raindrop size distribution |
| $z$ | Height |
| $R_{o}$ | Initial raindrop radius |
| $IPD$ | Inter-particle distance ($mm$) also $\lambda$ |
| $LWC$ | Liquid Water Content ($gm^{-3}$) |
| $Q$ | Magnitude of the raindrop source linked to the cloud auto-conversion rate |
| $(k_{1}^{'})$ | Modified auto-conversion rate ($s^{-1}$) |
| $F_{p}$ | Particle Froude Number |
| $\sigma_{z}$ | Plume standard deviation |
| $f_{r}$ | Probability of spotting a raindrop at a height $z$ |
| $R_{v}$ | Radius of line vortices (same as $\eta$) |
| $(M)$ | Rain mass ($gm^{-3}$) |
| $\nu$ | Viscosity of air ($m^{2}s^{-1}$) |
| $w$ | Updraught ($ms^{-1}$) |
| $V(r)$ | Velocity of the falling cloud droplets |
| $\Gamma$ | Vortex circulation strength ($m^{2}s^{-1}$) |
